# Somatic Mitochondrial–Nuclear DNA Transfer in Lymphoproliferative Disorders

**DOI:** 10.1002/jha2.70387

**Published:** 2026-09-15

**Authors:** James Peter Stewart, Nikos Darzentas, Miguel Alcoceba, Maria Eugenia Sarasquete, Anthony V. Moorman, Mark Catherwood, Binaya Regmi, Christiane Pott, Anton W. Langerak, David Gonzalez

**Affiliations:** ^1^ Johnston Centre for Cancer Research Queens University Belfast Belfast UK; ^2^ Department of Hematology University Hospital Schleswig‐Holstein Kiel Germany; ^3^ Cancer Research Center–IBMCC (USAL‐CSIC) University Hospital of Salamanca (HUS/IBSAL), CIBERONC Salamanca Spain; ^4^ Translational and Clinical Research Institute Newcastle University Newcastle upon Tyne UK; ^5^ Belfast City Hospital Belfast UK; ^6^ Department of Immunology, Laboratory Medical Immunology, Erasmus MC University Medical Center Rotterdam Rotterdam the Netherlands

## Abstract

**Introduction:**

Somatic mitochondrial–nuclear DNA transfer (SMNT) is a process by which mitochondrial DNA (mtDNA), of varying sizes, integrate into the nuclear genome and has been previously reported in solid tumours.

**Methods:**

EuroClonality‐NGS DNA Capture sequencing data from 755 lymphoid malignancies and 59 lymphoid cell lines were analysed for SMNT‐associated structural variants.

**Results:**

Five malignancies (0.66%) harboured SMNTs, predominantly involving *IGH* genes, with one *PTEN* gene disruption identified. Breakpoint features supported non‐homologous end joining‐mediated integration.

**Conclusion:**

SMNTs are rare but recurrent events in lymphoid malignancies and may represent stable clonal markers for minimal residual disease monitoring.

**Trial Registration:**

The authors have confirmed clinical trial registration is not needed for this submission.

1

To the Editor,

Clonal lymphoproliferative disorders (LPD) are lymphoid expansions arising from a single malignant cell which, in most cases, contain clonal immunoglobulin (IG) and/or T cell receptor (TR) rearrangements [1]. To achieve a diverse repertoire of IG/TR receptors, the IG/TR variable regions undergo a rearrangement process called somatic recombination, involving the variable (V), diversity (D) and joining (J) genes. Somatic recombination is mediated by the recombination‐activating gene (RAG) enzymes, which recognise recombination signal sequences that flank all V, D and J genes leading to the generation of double‐stranded breaks (DSBs) and excision of the intervening sequence. As an exemplar, DSBs in the IG heavy (IGH) chain are repaired using the non‐homologous end joining (NHEJ) pathway leading to one IGHV gene segment recombining with one of 27 IGHD gene segments and one of 6 IGHJ gene segments to form the variable domain [2]. Detection of clonal IG/TR rearrangements, achievable by multiple methods, provides relevant clinical and scientific information [1, 3, 4].

A hallmark of B/T cell malignancies are recurrent chromosomal translocations which often show evidence of occurring at the IG/TR loci during V(D)J recombination or class switch recombination [5, 6, 7]. Translocations require DSBs on both chromosomes and in LPDs this is typically caused by RAG or activation‐induced cytidine deaminase (AID) complexes with most DSBs repaired using NHEJ [8]. These processes highlight somatic recombination at the IG/TR loci is susceptible to formation of structural variants (SV).

Somatic mitochondrial–nuclear DNA transfer (SMNT) is a process by which mitochondrial DNA (mtDNA), of varying sizes; integrate into the nuclear genome [9, 10]. SMNT was reported in 10/559 (1.8%) primary cancers using whole genome sequencing (WGS), with most integrations showing evidence of NHEJ [10]. The pan‐cancer analysis of whole genomes consortium reported SMNT in 55/2658 (2.1%) cancers from across 38 tumour types using WGS [9]. SMNT frequency ranged from 0% (including blood cancers) to 16% in human epidermal growth factor receptor 2‐positive breast cancers. SMNT integration sites were associated with regions involved in translocations, inversions or large deletions [9, 10]. Recently, WGS analysis identified SMNT in 1/72 (1.4%) LPD cases with the mtDNA insertion occurring on Chromosome 4 [11]. WGS bioinformatic pipelines are unsuitable for the analysis of IG/TR regions due to large numbers of highly homologous V, D and J genes, which could potentially lead to an underestimation of SMNTs at IG/TR regions [12]. Within the context of LPD, we hypothesised SMNT could occur within IG/TR regions as (i) they undergo somatic recombination, (ii) are frequently the site of other SVs such as chromosomal translocations and (iii) somatic recombination and SMNT are both considered to utilise NHEJ for DSB repair.

We previously validated the EuroClonality‐NGS DNA Capture (EuroClonality‐NDC) Panel, which detects clonal IG/TR rearrangements, translocations, copy number variation (CNV) and somatic mutations in LPDs [13]. The EuroClonality‐NDC does not include probes for mtDNA; however, as a capture‐based target enrichment assay, it can identify novel SVs by capturing one of the partner genes with more than 95% sensitivity and specificity [13]. We therefore examined NGS data generated using the EuroClonality‐NDC assay in 755 lymphoid malignancies and 59 Human B (*n* = 30) and T (*n* = 29) lymphoid cell lines [13, 14]. Following alignment to GRCh38/hg38, the resulting Binary Alignment Map (BAM) files were visualised using Integrative Genomics Viewer and SVs identified by examining chimeric reads and/or soft clipped reads, which aligned to both the mitochondrial genome and a different chromosome [15].

Figure 1A details the 21 LPD subtypes represented in our cohort with 5 of 755 (0.66%) samples identified with evidence of SMNT with no detectable SMNTs in 59 human lymphoid cell lines. The five samples included two chronic lymphocytic leukaemias (CLL), one plasma cell myeloma (PCM), one mantle cell lymphoma (MCL) and one B‐cell acute lymphoblastic leukaemia (B‐ALL). Samples exhibited SMNT occurring at the *IGH* locus (*n* = 4, 80%) and at the *PTEN* gene (*n* = 1, 20%).

**FIGURE 1:**
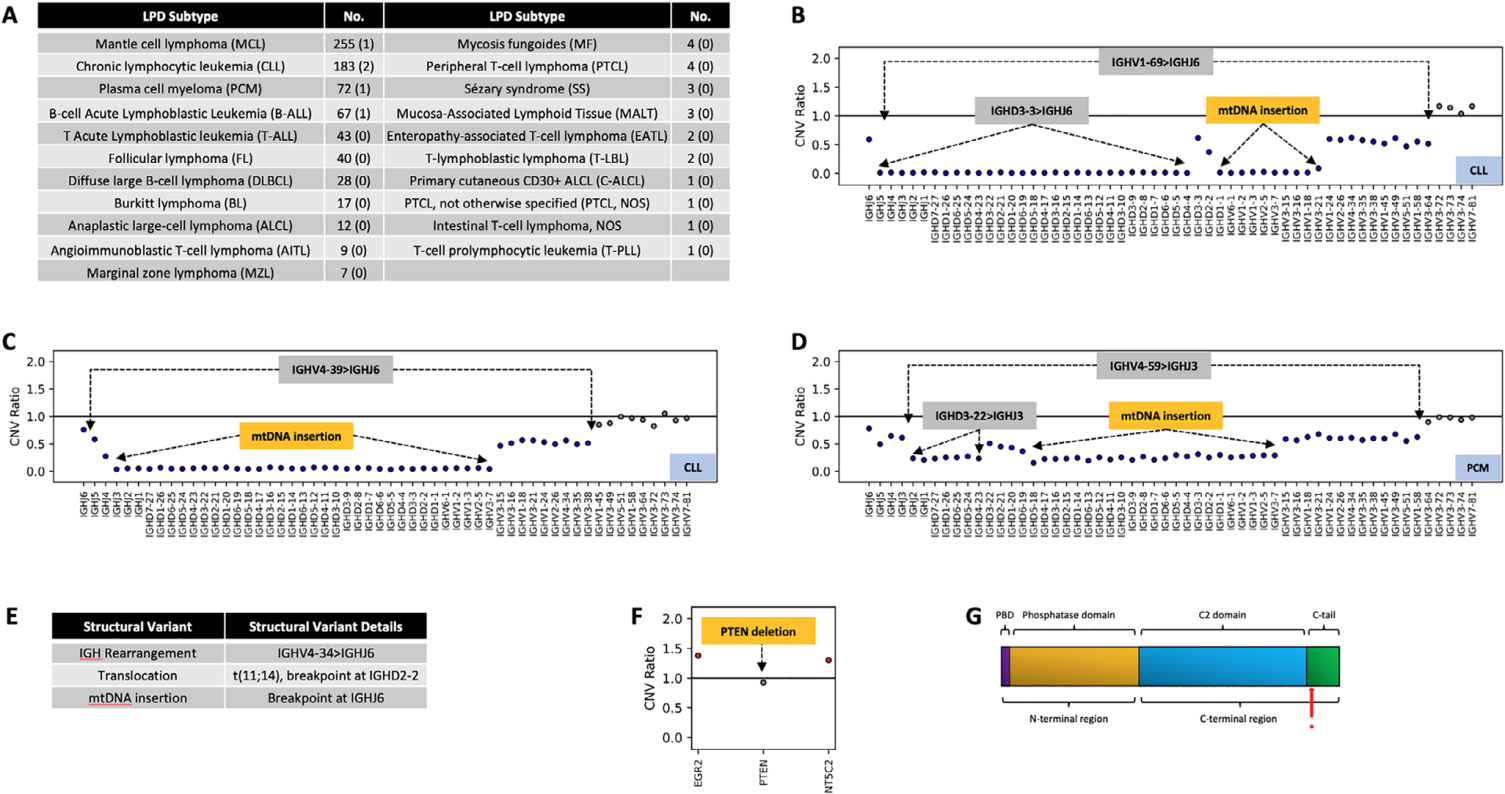
Summary of LPDs studied and examination of insertion points within nuclear DNA for mtDNA in 5 SMNT positive LPD samples. (A) Number of samples within each disease entity included in the study; the number in parentheses indicating cases showing an SMNT event. (B–D) Summary of the CNV ratio for the immunoglobulin heavy (IGH) region in samples with tumour infiltration > 20% and an SMNT event at the IGH locus. Disease entity is shown in the light blue shaded box with the rearrangements identified by the ARResT/Interrogate (grey shaded box) and mtDNA insertion points (yellow shaded box) highlighted to show agreement with coverage drops on the CNV plot. (E) Summary of SVs at the IGH region in the MCL sample with a tumour infiltration < 20%. (F) Summary of the CNV ratio for the *PTEN* gene in an ALL sample with an SMNT event at the *PTEN* locus. *EGR2* (10q21.3) and *NT5C2* (10q24.32‐q24.33) which are proximal and distal to the *PTEN* gene are shown for comparison. (G) Schematic representation of the PTEN protein indicating the location of the breakpoint (highlighted by a red arrow and asterisk) for the SMNT insertion in the *PTEN* gene.

To determine how SMNTs occur at the *IGH* locus while still enabling generation of a productive IGHV–IGHD–IGHJ rearrangement, we examined rearrangements, translocations and CNVs reported by the EuroClonality‐NDC. Figure 1B–D is graphical outputs displaying the CNV ratio, calculated by dividing the coverage depth of an individual tumour sample by the mean coverage depth value calculated from a pool of unmatched normal samples, for IGHV, IGHD and IGHJ genes captured by the EuroClonality‐NDC. Grey shaded boxes overlayed on the CNV plot indicate rearrangements reported by the EuroClonality‐NDC bioinformatics pipeline, ARResT/Interrogate [13].

In one CLL sample, which underwent sequencing on two separate occasions, a productive IGHV–IGHD–IGHJ rearrangement involving IGHJ6 and IGHV1‐69 and an IGHD–IGHJ rearrangement involving IGHD3‐3 and IGHJ6 were reported. Rearrangements were confirmed by corresponding drops in coverage of the intervening IGHV, IGHD and IGHJ gene segments on the CNV plot (Figure 1B). An SMNT with breakpoints at IGHV3‐23 and IGHD2‐2 was identified from the BAM file, which based on inspection of the CNV plot, led to deletion of the intervening IGHD and IGHV gene segments (Figure S1). The other CLL sample had a rearrangement involving IGHJ6 and IGHV4‐39 with confirmation by analysis of CNV data (Figure 1C). Inspection of the BAM file, identified SMNT breakpoints at IGHJ4 and IGHV3‐13 with the CNV plots demonstrating deletion of the intervening IGHD and IGHV gene segments (Figure S2).

Rearrangement and CNV data for the PCM sample were concordant, showing the presence of IGHV–IGHD–IGHJ and IGHD–IGHJ rearrangements with no translocations detected (Figure 1D). Analysis of the BAM file showed a SMNT breakpoint at IGHD6‐19, presumably on the same chromosome as the IGHD–IGHJ rearrangement (Figure S3). We did not detect the second breakpoint on the *IGH* locus although from inspection of the CNV plot the drop in coverage occurs between IGHV3‐7 and IGHV3‐15. This region has a recognised polymorphism (IGHV1‐8, IGHV3‐9 and IGHV2‐10) and a number of pseudogenes not covered by the EuroClonality‐NDC.

The MCL sample had a tumour infiltration of 4.5%, which is below the reported sensitivity of the CNV pipeline [13]. The *t*(11;14) translocation which occurs in > 95% of MCL cases was detected by FISH and confirmed by EuroClonality‐NDC (Figure 1E). An IGHV–IGHD–IGHJ rearrangement involving IGHV4‐34 and IGHJ6 was also identified. From BAM file analysis an SMNT insertion was located at IGHJ6 which was absent in the paired germline sample indicating SMNT occurred during tumorigenesis (Figure S4). The final sample (B‐ALL) showed evidence of a breakpoint within the *PTEN* gene (Figure S5). *EGR2* (10q21.3) and *NT5C2* (10q24.32) which are located proximal and distal to *PTEN* showed an elevated CNV ratio of 1.38 and 1.30 respectively, indicating an increased copy number of 10q. The *PTEN* gene (10q23.31) had a ratio of 0.92 implying a region of *PTEN* was deleted during the SMNT event (Figure 1F). The characterised breakpoint resides at codon 347 in Exon 9 and would potentially disrupt the C‐terminal tail domain (Figure 1G).

All 7 breakpoints identified in the 5 SMNT positive cases were examined in detail (Figure 2A). In two CLL cases, where both breakpoints involved in the SMNT were characterised, the mtDNA insert sizes were calculated as 141 and 645 bp. Insert sizes calculated from two publicly available datasets showed fragments of the 16569 bp mtDNA genome ranged from 2 to 16616 bp in 114 evaluable cases. We noted non‐template nucleotide insertions at the breakpoint which ranged from 3–51 bp in length. There was limited evidence of microhomology but in LPDs this is observed less frequently in chromosomal translocations compared to other NHEJ events [8]. SMNT breakpoints identified in our LPD cohort clustered in two discrete areas of the mtDNA genome with four breakpoints located within mtDNA positions 39–346 and three breakpoints occurring between 15940 and 15998. We plotted mtDNA breakpoint data from two publicly available datasets alongside our breakpoint data (Figure 2B). A disproportionate number of breakpoints were noted in the D‐loop region (16106‐104) of the mitochondrial genome with all characterised breakpoints identified in LPDs flanking this region.

**FIGURE 2:**
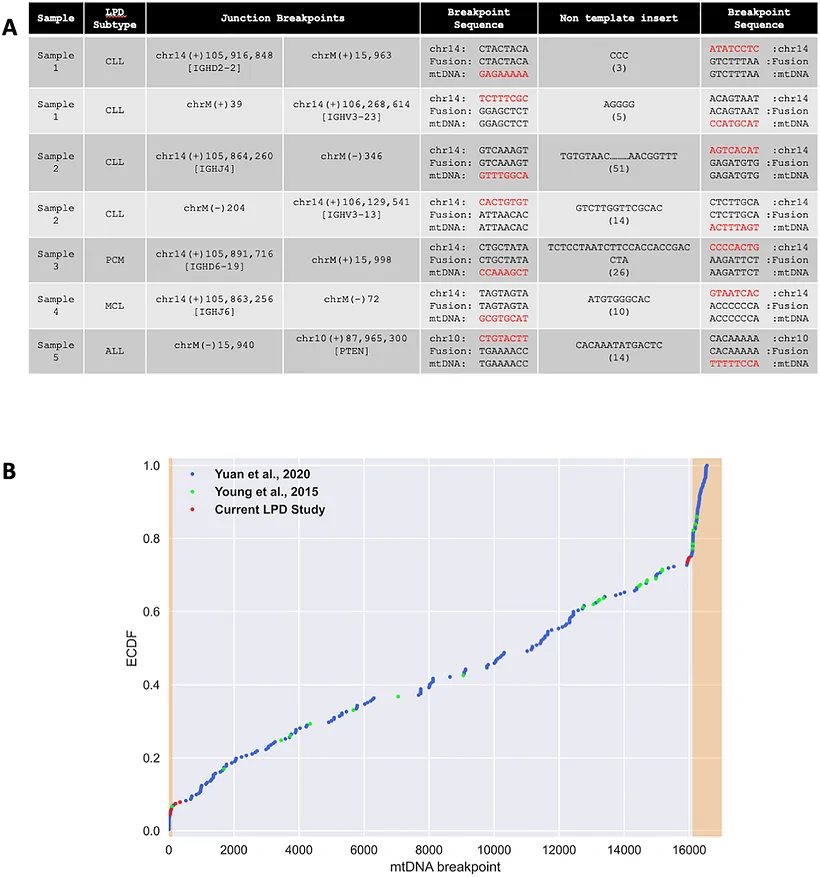
Assessment of breakpoint sequence and examination of mtDNA breakpoints in five positive SMNT LPD samples. (A) Characterisation of the seven breakpoints identified across the five positive SMNT LPD samples which indicates specific breakpoints on nuclear and mitochondrial DNA and size of non‐template insert DNA. The fusion sequence indicates the consensus sequence from reads spanning the breakpoint sequence. Mitochondrial DNA reference sequences, termed chrM, are from the GRCh38 assembly. (B) Empirical cumulative distribution function (ECDF) plot of mtDNA breakpoints from this study (red), Ju et al. [10] (green) and Yuan et al. [9] (blue). The orange shaded box indicates the D‐loop region.

Most SMNT insertions (80%) occurred at the *IGH* locus and involved an IGHV, IGHD or IGHJ gene segments, rather than a switch region, signifying SMNT occurs early in lymphomagenesis. While no SMNT events occurred at TR loci, regions also which undergo somatic recombination and are susceptible to formation of SVs such as translocations, only 83/755 (11.0%) of samples sequenced were of T‐cell origin. The SMNT insertions at the *IGH* locus did not impact on generation of a productive IG or presence of the *t*(11;14) translocation, considered to be the primary oncogenic event in MCL. Furthermore, the nuclear mtDNA fragments we characterised would not lead to insertion/expression of intact mtDNA genes. Using our data in combination with previous WGS studies, which identified no SMNT events in LPDs, SMNT events could be largely considered to be an opportunistic event arising mechanistically from the presence of mtDNA in the nucleus during the formation/repair of DSBs required for somatic recombination of the IGH/TR loci [9]. SMNT events leading to disruption of nuclear genes are rare and we identified only one SMNT insertion, which occurred within Exon 9 of the *PTEN* gene, leading to disruption of the tumour suppressor gene. While potentially not being an oncogenic driver, as SMNT occurs early in lymphomagenesis it may provide specific and stable clonal markers for MRD monitoring.

## Author Contributions

J.P.S. and D.G. designed the experiments, wrote the manuscript, contributed to molecular studies and performed data analysis. N.D developed computational tools, performed bioinformatic analysis and contributed to manuscript revision. M.A., M.E.S., B.R. contributed to molecular studies, data analysis and manuscript revision. A.V.M., M.C., C.P. and A.W.L. provided key scientific insights and/or provided samples and data and contributed to manuscript revision.

## Funding

The EuroClonality‐NGS Working Group is an independent scientific subdivision of EuroClonality that aims at innovation, standardisation and education in the field of diagnostic clonality analysis. The revenues of the previously obtained patent (PCT/NL2003/000690), which is collectively owned by the EuroClonality Foundation and licensed to InVivoScribe, are exclusively used for EuroClonality activities, such as for covering costs of the Working Group meetings, collective Work Packages and the EuroClonality Educational Workshops.

## Ethics Statement

This manuscript was developed from sequencing results arising from a retrospective analytical validation of a new method to detect clinically relevant alterations in comparison to current clinical standards (https://doi.org/10.1182/bloodadvances.2020004056). The specimens used were surplus DNA to diagnostic purposes and each participating centre followed their internal institutional policies for quality improvement studies.

## Conflicts of Interest

M.A. has received honoraria from Janssen and AstraZeneca, and non‐financial support ‐ event attendance ‐ from Janssen, Abbvie and Roche. A.W.L. has received contract research support from Roche‐Genentech, Gilead and Janssen. A.V.M. has received honoraria from Amgen. D.G. is founder and director of Univ8 Genomics and has received honoraria, consultance and/or research funding from Roche, AstraZeneca, Novartis, Eli Lilly, Janssen, Incyte and Illumina.

## Supporting information




**Supporting File 1**: jha270387‐sup‐0001‐figureS1.png

**Supporting File 2**: jha270387‐sup‐0001‐figureS2.png

**Supporting File 3**: jha270387‐sup‐0001‐figureS3.png

**Supporting File 4**: jha270387‐sup‐0001‐figureS4.png

**Supporting File 5**: jha270387‐sup‐0001‐figureS5.png

## Data Availability

The specimens used were surplus DNA to diagnostic purposes and patients were not routinely requested to consent for publication of their sensitive data such as DNA sequence.
